# Supplementary material for: Genomic Characterization of a Dog-Mediated Rabies Outbreak in El Pedregal, Arequipa, Peru
Source: bioRxiv. 2024 Aug 22:2024.08.21.608982. Preprint. [Version 1] doi: 10.1101/2024.08.21.608982 (PMC11370554; doi:10.1101/2024.08.21.608982)
Supplement: 1 [file NIHPP2024.08.21.608982v1-supplement-1.pdf]

bioRxiv preprint doi: https://doi.org/10.1101/2024.08.21.608982; this version posted August 22, 2024. The copyright holder for this preprint (which was not certified by peer review) is the author/funder, who has granted bioRxiv a license to display the preprint in perpetuity. It is made available under aCC-BY 4.0 International license.

Supplementary files

bioRxiv preprint doi: https://doi.org/10.1101/2024.08.21.608982; this version posted August 22, 2024. The copyright holder for this preprint (which was not certified by peer review) is the author/funder, who has granted bioRxiv a license to display the preprint in perpetuity. It is made available under aCC-BY 4.0 International license.

S1 Table. Sequencing and epidemiology details of newly sequenced rabies virus sequences used in this study.

NCBI accession	Isolate ID	Case ID	NGS platform	Library type	Virus sample set	NGS run	Mapped reads	Mean (stdev) coverage	Sequence length*	Sample Collection Date	Outbreak place	Region	Province	District
PP965373	1101786	-	Illumina	Metagenomic	1	illumina-1	173909	2987 (741)	11869	26-Mar-11	Puno	Puno	Azangaro	Caminaca
KU938752*	1101787	-	Illumina	Metagenomic	1	illumina-1	731825	6536 (1471)	11874	3-Mar-11	Puno	Puno	Azangaro	Caminaca
KU938829*	1203037	-	Illumina	Metagenomic	1	illumina-1	12966	237 (169)	11842	8-May-12	Puno	Puno	Puno	Atuncolla
PP965367	107_2018	-	Illumina	Metagenomic	2	Illumina-2	1955	47 (52)	10500	25-Mar-18	Arequipa	Arequipa	Arequipa	Cerro Colorado
PP965364	107_2021	6	Nanopore	Amplicon	3	nano-1	360339	13521 (7447)	10563	20-Mar-21	El Pedregal	Arequipa	Caylloma	Majes
PP965360	121_2019	-	Illumina	Metagenomic	2	Illumina-2	1236	30 (12)	11791	22-Apr-19	Arequipa	Arequipa	Arequipa	Cerro Colorado
PP965366	126_2021	-	Nanopore	Amplicon	3	nano-2	41076	1826 (726)	11816	31-Mar-21	Arequipa	Arequipa	Arequipa	Cerro Colorado
PP965361	145_2022	-	Nanopore	Amplicon	3	nano-2	69588	2757 (1405)	11827	9-Jun-22	Arequipa	Arequipa	Arequipa	Cerro Colorado
PP965351	147_2021	7	Nanopore	Amplicon	3	nano-1	372481	14426 (6599)	10565	19-Apr-21	El Pedregal	Arequipa	Caylloma	Majes
PP965365	147_2022	-	Nanopore	Amplicon	3	nano-2	35162	1666 (1144)	10752	10-Jun-22	Arequipa	Arequipa	Arequipa	Yura
PP965369	158_2022	-	Nanopore	Amplicon	3	nano-2	26045	1500 (545)	11825	21-Jun-22	Arequipa	Arequipa	Arequipa	Jacobo Hunter
PP965371	172_2022	-	Nanopore	Amplicon	3	nano-2	83048	3167 (1618)	11812	8-Jul-22	Arequipa	Arequipa	Arequipa	Cayma
PP965368	173_2022	-	Nanopore	Amplicon	3	nano-2	60646	2606 (1257)	11819	12-Jul-22	Arequipa	Arequipa	Arequipa	Jacobo Hunter
PP965362	182_2019	-	Illumina	Metagenomic	2	Illumina-2	1044	25 (16)	11273	16-Jun-19	Arequipa	Arequipa	Arequipa	Alto Selva Alegre
PP965344	202_2021	8	Nanopore	Amplicon	3	nano-1	112087	7031 (1900)	10563	14-Jun-21	El Pedregal	Arequipa	Caylloma	Majes
PP965347	219_2022	-	Nanopore	Amplicon	3	nano-2	36608	2472 (878)	11822	31-Aug-22	Arequipa	Arequipa	Arequipa	Characato
PP965374	231_2021	-	Nanopore	Amplicon	3	nano-2	28192	1405 (515)	11801	15-Jul-21	Arequipa	Arequipa	Arequipa	Cerro Colorado
PP965345	236_2022	12	Nanopore	Amplicon	3	nano-1	758917	25058 (17387)	9755	7-Sep-22	El Pedregal	Arequipa	Caylloma	Majes
PP965363	250_2021	9	Nanopore	Amplicon	3	nano-1	350173	14068 (5935)	10565	10-Aug-21	El Pedregal	Arequipa	Caylloma	Majes
PP965354	251_2021	10	Nanopore	Amplicon	3	nano-1	115094	7740 (2039)	10565	10-Aug-21	El Pedregal	Arequipa	Caylloma	Majes
PP965359	30_2021	-	Nanopore	Amplicon	3	nano-2	50742	2183 (936)	11821	5-Feb-21	Arequipa	Arequipa	Arequipa	Yura
PP965357	31_2021	-	Nanopore	Amplicon	3	nano-2	71634	2593 (1262)	11820	8-Feb-21	Arequipa	Arequipa	Arequipa	Cerro Colorado
PP965349	34_2021	1	Nanopore	Amplicon	3	nano-1	148926	7481 (2403)	10565	10-Feb-21	El Pedregal	Arequipa	Caylloma	Majes
PP965355	3553_2010	-	Illumina	Metagenomic	1	Illumina-2	3848	95 (52)	11904	2010	Puno	Puno	Azangaro	
PP965352	375_2017	-	Illumina	Metagenomic	2	Illumina-2	3545	88 (82)	11893	23-Nov-17	Arequipa	Arequipa	Arequipa	Mariano Melgar
PP965353	40_2021	-	Nanopore	Amplicon	3	nano-2	9237	393 (169)	11826	15-Feb-21	Arequipa	Arequipa	Arequipa	Cerro Colorado
PP965343	52_2021	2	Nanopore	Amplicon	3	nano-1	47951	3833 (1027)	10565	24-Feb-21	El Pedregal	Arequipa	Caylloma	Majes
PP965358	56_2021	-	Nanopore	Amplicon	3	nano-2	70889	2336 (1445)	11580	27-Feb-21	Arequipa	Arequipa	Arequipa	Yura
PP965348	560_2015	-	Illumina	Metagenomic	2	Illumina-2	2106	52 (30)	11884	7-Sep-15	Arequipa	Arequipa	Arequipa	Mariano Melgar
PP965346	61_2021	-	Nanopore	Amplicon	3	nano-2	70413	2947 (1222)	11826	1-Mar-21	Arequipa	Arequipa	Arequipa	Cerro Colorado
PP965356	68_2021	3	Nanopore	Amplicon	3	nano-1	245893	10765 (4169)	10563	3-Mar-21	El Pedregal	Arequipa	Caylloma	Majes
PP965350	71_2021	4	Nanopore	Amplicon	3	nano-1	328914	13188 (5130)	10564	6-Mar-21	El Pedregal	Arequipa	Caylloma	Majes
PP965370	73_2021	5	Nanopore	Amplicon	3	nano-1	42452	3467 (812)	10564	6-Mar-21	El Pedregal	Arequipa	Caylloma	Majes
PP965372	90_2021	-	Nanopore	Amplicon	3	nano-2	45757	1838 (825)	11826	13-Mar-21	Arequipa	Arequipa	Arequipa	Yura

*Genbank records updated
